# Using a Balloon-Launched Unmanned Glider to Validate Real-Time WRF Modeling

**DOI:** 10.3390/s19081914

**Published:** 2019-04-23

**Authors:** Travis J. Schuyler, S. M. Iman Gohari, Gary Pundsack, Donald Berchoff, Marcelo I. Guzman

**Affiliations:** 1 Department of Chemistry, University of Kentucky, Lexington, KY 40506, USA; travis.schuyler@uky.edu; 2 Director of SaaS Development, TempoQuest Inc., Boulder, CO 80303, USA; iman.gohari@tempoquest.com; 3 Stratodynamics Aviation Inc., Kenilworth, ON N0G 2E0, Canada; gpundsack@stratodynamics.ca; 4 TruWeather Solutions, Reston, VA 20194, USA; don.berchoff@truweathersolutions.com

**Keywords:** UAV, UAS, glider, meteorology, weather, WRF, ARW, GFS, model, validation, drones, balloon, radiosonde, temperature, pressure, relative humidity

## Abstract

The use of small unmanned aerial systems (sUAS) for meteorological measurements has expanded significantly in recent years. SUAS are efficient platforms for collecting data with high resolution in both space and time, providing opportunities for enhanced atmospheric sampling. Furthermore, advances in mesoscale weather research and forecasting (WRF) modeling and graphical processing unit (GPU) computing have enabled high resolution weather modeling. In this manuscript, a balloon-launched unmanned glider, complete with a suite of sensors to measure atmospheric temperature, pressure, and relative humidity, is deployed for validation of real-time weather models. This work demonstrates the usefulness of sUAS for validating and improving mesoscale, real-time weather models for advancements toward reliable weather forecasts to enable safe and predictable sUAS missions beyond visual line of sight (BVLOS).

## 1. Introduction

The use of unmanned aerial vehicles (UAVs) as sensor platforms for meteorology and atmospheric monitoring has gained significant popularity within the scientific community over the last few years. The ability to collect data with high spatiotemporal resolution allows for atmospheric studies that were previously much less practical [1]. For example, field experiments with small unmanned aerial systems (sUAS) show it is possible to accurately measure atmospheric temperature [2], pressure, relative humidity [3], and wind speed/direction [4]. This raw data can be used to derive more interesting information, such as potential temperature [5], absolute humidity [3], wind turbulence [6], sensible heat flux [7], convection initiation [8], and boundary layer transitions [9]. Overall, this data allows for a better understanding of how atmospheric conditions affect weather patterns and lead to more informed meteorological predictions [10].

Data collected during flights with sUAS can also provide information for advanced weather models [11], such as the widely utilized weather research and forecasting (WRF) model, developed in the 1990s. WRF models can be generated based on real measured atmospheric conditions or idealized conditions. The WRF model assimilates data from thousands of registered sites across the globe and integrates them into a model that allows for parallel computation and system extensibility [12]. WRF models are used extensively for real-time forecasting and meteorology throughout the world. These models use next-generation NVIDIA graphical processing units (GPUs) to predict atmospheric temperature, relative humidity, pressure, and wind in near real time, making it very useful for comparison to UAS data collected in situ [13]. Combining both modeled and sUAS measured data to enhance meteorological predictions is a necessary area that requires further exploration.

Different methods to improve weather predictions from in situ measurements have been reported in literature. Among them, a manned Lockheed C-130 evaluated the ability of mesoscale forecast models to predict atmosphere–ice–ocean interactions as seasons evolve [14]. NASA has used a full scale unmanned aircraft, the Global Hawk (GH) jet, for a variety of stratospheric missions, i.e., flying at approximately 20 km altitude for >31 h, over an extended trajectory of >20,372 km [15]. Dropsonde missions carried out by the GH (capable of carrying payloads of 681 kg) have provided pressure, temperature, and relative humidity data with 6 m resolution in altitude [16], which, combined with information from instruments in satellites, has been used to improve weather forecasts of tropical cyclones [17]. Despite the success of the GH-driven dropsonde missions, the large construction and operation costs of the GH jet make their availability to most research groups highly limited. Similarly, the disadvantages of operating the C-130 for research applications include the high cost of deployment and crew operation as well as the limited spatiotemporal resolution at the high airspeeds traveled.

Smaller scale UAVs, such as the Small Unmanned Meteorological Observer (SUMO), completed meteorological measurements in the atmospheric boundary layer (ABL) carrying <1 kg of payloads, flying <4 km altitude for 30 min [18]. Furthermore, the Small Multifunction Autonomous Research and Teacher Sonde (SMARTSonde) was built to research ABL structure and inspect it during an evening boundary layer transition [19]. The creation of the Aerosonde with a Vaisala payload [20] enabled UAV measurements comparable to traditional radiosondes, but they are still susceptible to winds. Another platform of potential interest, when launched by a balloon, is the DataHawk airborne system that could potentially gather temperature and relative humidity profiles up to 9 km altitude [21]. However, available measurements of temperature and relative humidity only confirmed the usefulness of the DataHawk at up to 3.6 km altitude in flights lasting 20 min [21]. Although smaller scale UAVs, i.e., the SUMO, SMARTSonde, Aerosonde, and DataHawk, are useful for the specific objectives discussed above, they are unable to fly the long time and high altitude needed to examine a mesoscale process, limiting their application for WRF model analysis evaluation. The combination of a ground-based and unmanned aircraft system for weather measurements [22] could only be used to validate measurements 10–15 m above ground level (AGL), not providing enough data for WRF model validation. Lastly, rotary wing UAVs have been used for similar meteorological research objectives. When a hexarotor UAV is deployed to measure wind vector profiles up to 1000 m AGL, its flight time is limited to only 20–25 min [23] in the close vicinity.

We propose a solution to this problem by using balloon-launched unmanned gliders. The advantages of using a glider in this work versus the possibility of employing a propeller driven aircraft are: (1) the turbulent air flow behind and around the propeller can potentially corrupt the sensor readings, for example, by affecting the temperature of airflow at the sensor location. For this study, placement of the sensors or the inlets was selected where static pressure was expected. We must note that a valid alternative is to place the motors on the wing to free the nose of the aircraft with the sensors from any propeller effects, i.e., as offered by the TTwistor sUAS [24]; (2) a powered aircraft would be unable climb to this altitude without a significantly larger and energy intensive power package, which justifies the strong need to utilize a balloon for the climb portion of the flight. For this same reason, the powered DataHawk system launched by a balloon [21] demonstrated relatively short time temperature and relative humidity data collection up to 3.6 km altitude; (3) the use of a glider saves considerable energy to be consumed in other scientific operations, and leverages the weight that a motor and battery source demand for payload needs. Thus, the use of a glider decreases the balloon size and the amount of helium required, as compared to a propeller aircraft, and decreases the airframe size. Overall, this compelling reason is also supported by the relatively simple logistics and low cost needed for deployment. Based on the power source selected, the measurements provided by the glider always last longer than the time needed for the descending flight to reach the ground; (4) finally, the glider has an efficient airfoil, which enables a slow controlled descent, compared to pure parachute-borne descent devices. Similarly, the superior airfoil for the glider rather than for existing powered aircraft such as the TTwistor [24] results in a better aerodynamic efficiency.

In this work, we analyze the data collected on-board a balloon-launched unmanned glider called the HiDRON from Stratodynamics Aviation Inc. and compare it to WRF-modeled weather data (TempoQuest Inc., TruWeather Solutions) for the same time and position. The focus of this work is to correlate temperature, pressure, and relative humidity measurements with altitude during descending flights starting at 25 km. By analyzing these descent profiles, the work demonstrates the usefulness of sUAS, and particularly balloon-launched unmanned gliders, for atmospheric measurements for weather model validation. Therefore, the novelty of the work presented is centered in collecting high spatiotemporal resolution physical measurements up to 25 km altitude to validate the WRF model. In this initial stage, the work does not intend to improve the forecast performance. The overall goal of this combined field and computational study is to provide a proof of concept of how powerful sUAS measurements can be to validate weather predictions, i.e., up to 25 km altitude, and potentially enable the reevaluation of models for future improvements.

## 2. Materials and Methods

The construction of the unmanned glider employed, along with the sensor package for atmospheric monitoring, is addressed in Section 2.1. The high resolution, real-time WRF model (TempoQuest Inc., Boulder, CO, USA, and TruWeather Solutions, Renton, WA, USA) is described in Section 2.2.

### 2.1. Airframe and Instrumentation

**Figure 1:**
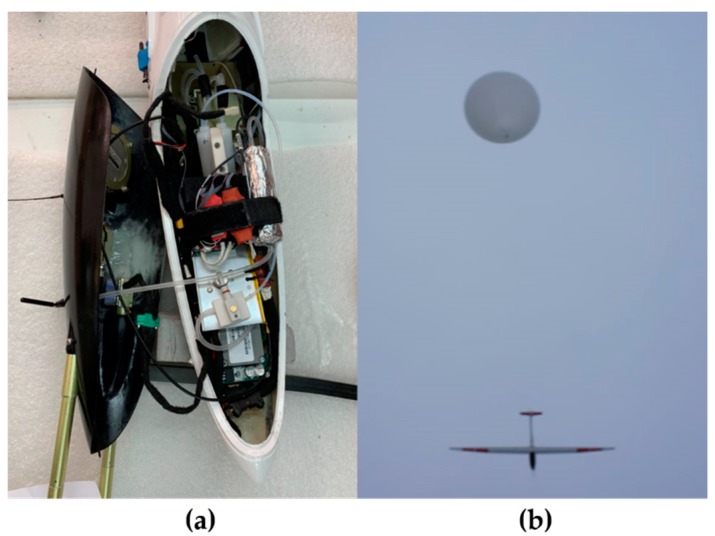
(**a**) The unmanned glider (HiDRON) fuselage, equipped with a vacuum-pumped inlet tube connected to a thermally insulated chamber containing sensors for temperature, pressure, and relative humidity. (**b**) The weather balloon brings the unmanned glider to the desired release altitude before releasing from the tail.

The unmanned glider, called the HiDRON (Stratodynamics Aviation Inc., Kenilworth, Canada), features a glider airframe with a high-quality airfoil and composite carbon fiber and fiberglass construction (Figure 1). Its wingspan is nearly 3.4 m. The aircraft is controlled by means of a rudder, elevator, ailerons, and flaps with servo drives. The avionics system includes navigation and autopilot components and is capable of autonomous and beyond visual line of sight (BVLOS) operations. Location-specific weather guidance has been recommended in work comparing sUAS flights with WRF predictions limited to 250 m AGL for a 100 km^2^ grid [24], which require relatively simple flight permissions. However, flying up to 25 km altitude AGL requires special authorization for flights beyond visual line of sight due to aerospace regulations. The location selected in Belarus provided a unique opportunity to complete the mission in the 6 h window and dedicated aerospace, while diverting other flight traffic from our sUAS. The autopilot system is reliable under cold temperatures, i.e., −60 °C at 25 km altitude, and maintains communication with the ground station. The HiDRON autopilot system utilized had been previously rated to operate well at −40 °C, and the system had been field tested up to 20 km altitude, while other commercial devices were only rated to −20 °C. In order to ensure communication was maintained with the ground station during this work, a two-axis high-gain tracking antenna was utilized at the ground station.

The HiDRON flies by the UAVOS AP10.3 automatic control system. Its total weight was 4.15 kg with the payload instrumentation. The system components consist of navigation, telemetry, GPS/GNSS, pitot tube, and pitot data processing units. Each component of the system has its own microcontroller and is connected in a distributed architecture, providing data processing and communication with other components within the Controller Area Network (CAN). The navigation unit processes commands and includes two 3-axis accelerometers, two 3-axis gyros, and one 3-axis magnetometer (compass). The telemetry unit processes the integrated 928 MHz radio link and controls the servos for the aircraft control surfaces and emergency parachute. The GPS/GNSS receiver includes a 3-axis accelerometer, a 3-axis gyro, and a 3-axis magnetometer. The pitot tube has static and dynamic ports, and can be heated to prevent icing and remove moisture. The pitot data processing unit provides the indicated airspeed.

To measure weather conditions during the flight, an iMET-XF (InterMet Systems Inc., Grand Rapids, MI, USA) was customized to fit inside the fuselage of the HiDRON. The temperature and relative humidity sensors were secured in a thermally isolated vacuum-pumped flow cell (50 mL min^−1^) that was connected to a static port tube protruding out of the nose of the glider. The overview of the fuselage containing the sensor package is pictured in Figure 1. Atmospheric pressure was measured on the iMET-XF circuit board. The temperature sensor was a negative temperature coefficient (NTC) bead thermistor and the relative humidity sensor was a capacitive HYT 271 sensor, both calibrated by InterMet Systems Inc. The practically perfect agreement between the predicted and measured pressure allowed the direct comparison of physical data to the WRF model without the need to calculate the potential temperature and absolute humidity. The iMET XF radiosonde was connected to the telemetry unit and data from the payload were sent to the ground station, along with the GPS/GNSS and other flight data at 10 Hz intervals. An auxiliary iMET SD data acquisition system was connected to the radiosonde board as a backup for the radiosonde data. Since the ground station was able to collect all the payload data and timestamp it with GPS/GNSS and other data, the backup data system was unnecessary for this configuration. The data compiled at the ground station enabled the observed payload data to be synchronized with the flight data to provide an accurate correlation to position, orientation, speed, acceleration, and other flight data.

### 2.2. Details of WRF Modeling

WRF is the Advanced Research WRF (ARW) modeling system that has been in development for the past decade (WRF-ARW V3 Users Guide [25]). The ARW is designed to be a flexible, state-of-the-art atmospheric simulation system that is portable and efficient on available parallel computing platforms. The ARW is suitable for use in a broad range of applications across scales ranging from meters to thousands of kilometers, including parameterization research, forecast research, real-time numerical weather prediction (NWP), and dynamical downscaling. The WRF model has a nesting capability and can accommodate multiple embedded nests, which are attractive for such studies.

To provide a real-time WRF modeling, an operational dynamical downscaling was performed using two inner domains out of three, in which mesoscale motions gradually scale down to terrain-resolving motions. Therefore, the present modelling explicitly resolves the convective-scale motions, which are necessary for an accurate forecast required by mission critical applications such as sUAS.

**Figure 2:**
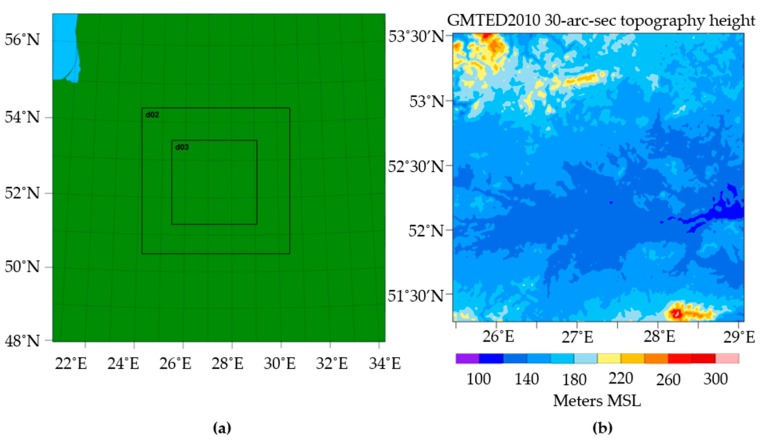
The geographical representation of the WRF model configuration: (**a**) The nested domains; (**b**) the terrain height over the innermost domain, i.e., d03.

Table 1 summarizes the model configuration of current operational dynamical downscaling modeling. The model runs using two-way-nested domains with horizontal grid spacings of 3 km and 1 km, while the outer 9 km domain is initialized and driven by Global Forecast System (GFS) data (Figure 2). The boundary conditions of the outer 9 km domain are updated every 3 h from GFS data, while the boundary conditions of the two-way-nested domains are taken from the outer 9 km domain. For the targeted validation time period of 16 December 2018, 12:00–6:00 UTC, the operational dynamical downscaling was initialized with GFS data from 15 December, 6:00, to provide an 18 h spin-up time for the WRF model [26]. During this spin-up time, the WRF model adjusts the initial conditions to be thermodynamically balanced, as well as the small-scale motions on the nested domains to be developed [26,27].

The WRF model datasets were visualized in the NCAR Command Language (NCL) environment every 0.1 km from 0.2 to 1 km and every 1 km from 2 to 25 km. The model data were generated for every hour of the sUAS’s scheduled flight window from 12:00 to 6:00. UTC. The modeled data were then matched to the HiDRON’s altitude at the correct time to ensure the most correct model data were used for analysis. The temperature, pressure, and relative humidity was extrapolated from the model data at the correct altitude, time, and location of the glider. The extrapolated data were then plotted alongside the continuous data collected by the glider on the descent profile.

## 3. Results and Discussion

A summary of the balloon launch and unmanned glider flight is provided in Section 3.1, and an analysis of the atmospheric temperature, pressure, and relative humidity measurements collected during the 25 km descent profiles is presented in Section 3.2. An example of the WRF models generated for the altitude slices is provided and discussed in Section 3.3. The atmospheric profiles of temperature, pressure, and relative humidity for the measurements and WRF model are plotted and analyzed in Section 3.4.

### 3.1. Balloon Launched Unmanned Glider

**Figure 3:**
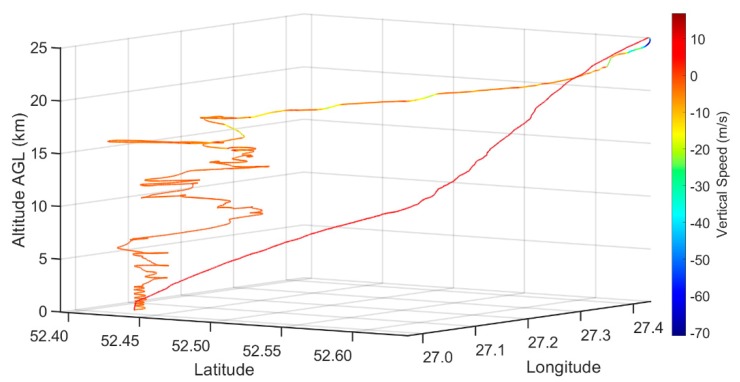
Trajectory of the rising balloon glider up to 25 km above ground level (AGL), followed by the launching and flight path of the glider on 16 December 2018 from 2:00 to 5:55 UTC. The line color depicts the vertical speed of the glider.

The balloon launch took place on 16 December 2018. The launch site for the 25 km AGL flight was 170 km south of Minsk, Belarus. The unmanned glider was launched with a 1500 g balloon filled with helium to 7.5 m^3^ (estimated), generating 6175 g of neck lift with a maximum theoretical height of 29.5 km. The anticipated vertical speed of the balloon was calculated to be at least 5 m s^−1^. The glider was attached to the balloon via a 10 m line to the glider’s tail with a redundant release mechanism to ensure a release within 50 m of the desired 25 km height. The balloon was released at 2:00 UTC, and reached 25 km altitude at approximately 3:00 UTC, at which point the glider was released from the balloon. At the release altitude, the glider was 36 km away from the launch site and glided back to the launch area, where it began loitering at an altitude of 18 km. The glider landed at the launch location at 5:55 UTC. Figure 3 illustrates the flight path of the glider from launch to landing. The color of the line represents the vertical speed of the glider. The telemetry and all on-board sensors were viewed live on the autopilot software.

The glider ascended with an average vertical speed of 7.5 m s^−1^, and a mean ground speed of 38.0 m s^−1^. The glider traveled 35.5 km from the launch location during the ascent in approximately one hour. Once released from the balloon, a maximum vertical speed of −70.9 m s^−1^ was achieved and nearly 2.5 G’s of force were placed on the unmanned glider as it pulled up from the initial balloon release. Once stabilized, the glider maintained an average ground speed between 46 and 47 m s^−1^ and an average vertical speed of −5.8 m s^−1^ until it reached a 15 km altitude. From 15 km to the tropopause, the ground speed slowed to an average of 27 m s^−1^ and an average vertical speed of −2.0 m s^−1^. From the tropopause until touchdown, the average ground speed was 14.8 m s^−1^ and the average vertical speed was −1.6 m s^−1^.

As expected, the vertical speed of the HiDRON varies with air density, and is greater at higher altitudes. The average vertical speed was −2 m s^−1^ from 24 km altitude to landing. These observations indicate the data resolution gathered with the glider was higher than a device descending with a parachute. Future work could attempt to further decrease the vertical speed by adjusting and tuning the autopilot controls. The glider uses the wind to increase the distance covered and, on this flight, profiled nearly 850 km^2^. The window for flight permissions ended at 6:00 UTC, so the flight time of the glider was shorter than the theoretical maximum flight time. It is estimated that with a maximum flight time of 6 h, nearly 2000 km^2^ can be covered under similar conditions.

### 3.2. Meteorological Profile Data from Balloon-Launched Unmanned Glider

**Figure 4:**
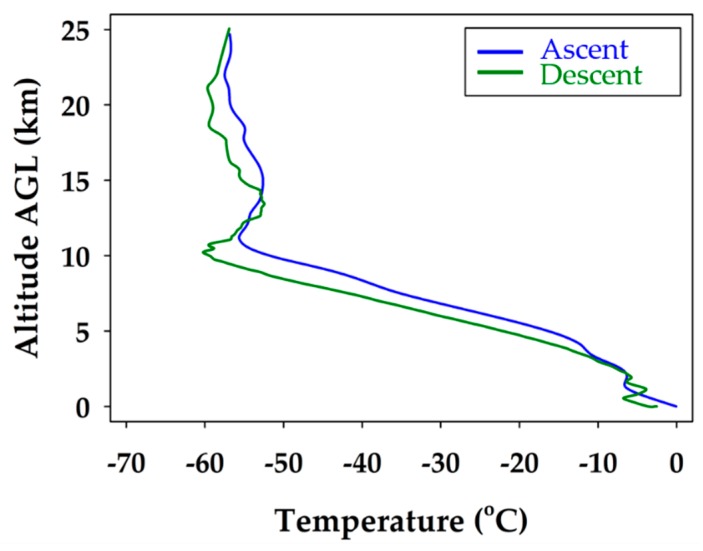
Air temperature profile during the ascent trajectory of the balloon glider (blue) and descent flight path of the unmanned glider (green). The boundary layer is observed in the lowest few hundred meters, the tropopause at about 10 km, and a typical temperature inversion is registered, showing a stable stratospheric layer.

The iMET-XF circuit board, integrated into the fuselage of the glider, was powered upon takeoff. This enabled the collection of temperature, pressure, and relative humidity during the flight. The full atmospheric profile for temperature is shown in Figure 4. The boundary layer in the lowest few hundred meters is observed, until about 2 km, where a typical lapse rate is measured. This trend continues until the tropopause at 10 km, and another temperature inversion is present, followed by a stable stratospheric layer from 15 to 25 km.

**Figure 5:**
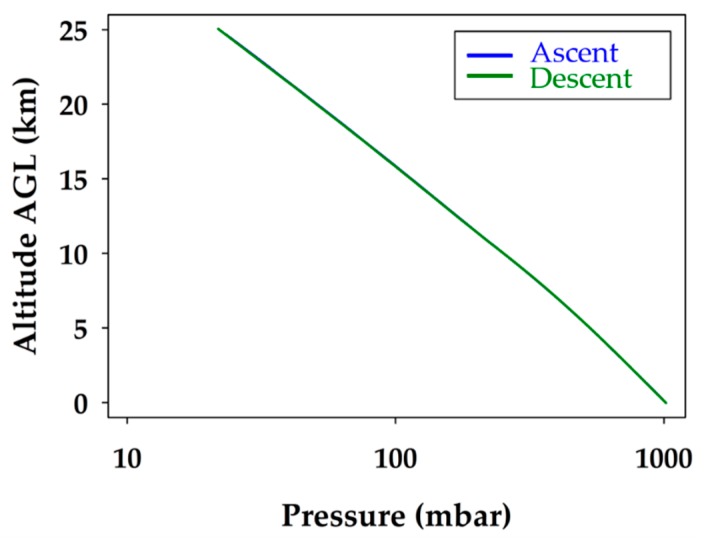
Atmospheric pressure profile during the balloon ascent (blue) and unmanned glider flight descent (green). The logarithmic pressure scale provides the expected linear relationship with altitude.

The atmospheric pressure profile is plotted in Figure 5. The observed atmospheric pressure profile is the expected linear relationship with altitude when plotted on a logarithmic pressure axis. As illustrated in Figure 5, the pressure did not change between the ascent and descent, as the lines overlap. This indicates that the environment inside the fuselage of the glider was representative of the air around it. It also shows that the static port worked effectively, as the pressure did not spike anywhere during the descent when speeds reached over 70 m s^−1^.

**Figure 6:**
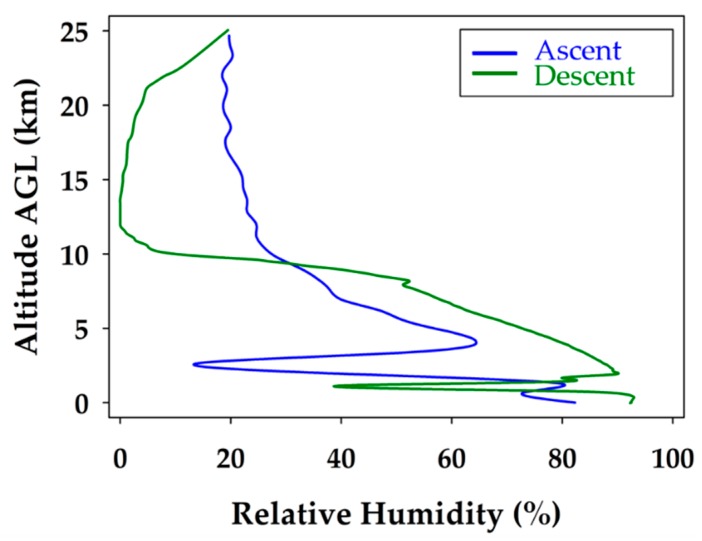
Relative humidity profile during the balloon ascent (blue) and unmanned glider descent (green). The break in the cloud layers at 3.5 and 2 km on the ascent and descent is observed. The decrease in relative humidity above the tropopause correlates with the drop in temperature measured.

The relative humidity profile during the ascent and descent of the glider flight is shown in Figure 6. The sharp drops in relative humidity clearly indicate a break in the cloud layers present on the ascent and descent at 3.5 and 2.0 km respectively. The tropopause is also evident in the relative humidity profile as a significant change in relative humidity is observed at the same altitude as the temperature inversion, 10 km. The biggest change in relative humidity occurred between the ascent and descent during the stratospheric portion of the flight. The cloud cover cleared during the descent portion of the stratospheric flight but converged again once below the tropopause.

### 3.3. WRF Modeling Data

**Figure 7:**
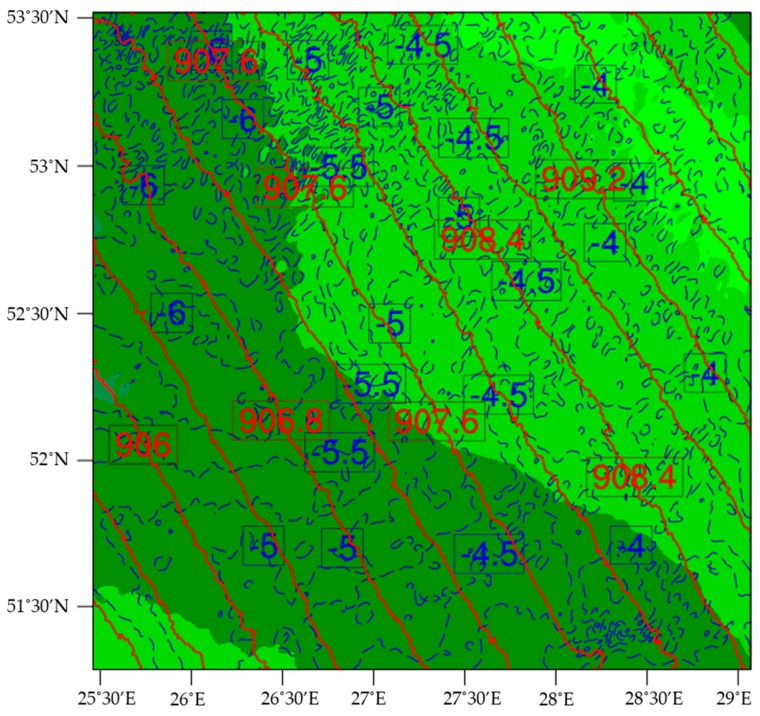
Example of weather simulation for 16 December 2018 at 1 km AGL (2:00 UTC). The simulations include atmospheric temperature (blue contour lines) reported in °C, pressure (red contour lines) reported in mbar, and relative humidity (background shading) ranging from 70% (light green) to 100% (dark green).

The raw simulation data received from TempoQuest Inc. and TruWeather Solutions were input into an NCL script and processed in the Linux NCL environment. The raw weather data were collected on 15 December 2018 at 6:00 UTC. TempoQuest Inc. ran simulations for every hour on 16 December 2018 from 12:00 UTC to 6:00 UTC, the provided test flight window for the glider. The NCL script was written to generate plots from 0.2 to 25 km, and altitude slices were taken every 0.1 km from 0.2 km to 1 km, and every 1 km from 2 km to 25 km. The plots were carefully selected at each altitude to match the time and position of the unmanned glider and the data were extrapolated accordingly. Figure 7 shows an example of the weather simulation graphic for 16 December 2018 at 2:00 UTC at 1 km. In these models, the blue lines represent the temperature contours, the red lines illustrate the pressure contours, and the background shading corresponds to the relative humidity.

At 1 km, the temperature ranges from −3.5 to −6.5 °C, with contours of 0.25 °C. The pressure ranges from 905.2 to 910.4 mbar by 0.4 mbar. The humidity is very high, nearing 90 to 100% over the region containing the glider’s takeoff location. In contrast, modeled conditions at the top of the 25 km profile are quite different. In the weather models at 25 km, temperatures have dropped to a range of −67 to −69 °C by 0.2 °C, and the pressure is down to a range of 26.70 to 27.08 mbar by 0.03 mbar. At this altitude, the relative humidity is less than 20%.

### 3.4. Evaluation of Measured and Modeled Data

The temperature, pressure, and relative humidity data from the weather simulation (for every altitude slice and at the appropriate time/location) were extrapolated and plotted, along with the measured data collected onboard the unmanned glider. The measured data are illustrated with the black line, and the red line corresponds to the modeled data. The modeled data are compared to the measured data during the descent because the unmanned glider was executing a loiter pattern, a more desirable flight path for accurately measuring atmospheric conditions. Because no previous high spatiotemporal resolution data covering the interval from 0 to 25 km altitude existed, the physical data collected directly validate the WRF model. For this purpose, a two-tailed T hypothesis test is carried out to evaluate the difference between the measurements and the WRF model at a 95% confidence level. If the calculated difference is greater than the pre-established tolerance, *p* = 0.05, the predicted altitude-dependent value by the model is considered unsound.

**Figure 8:**
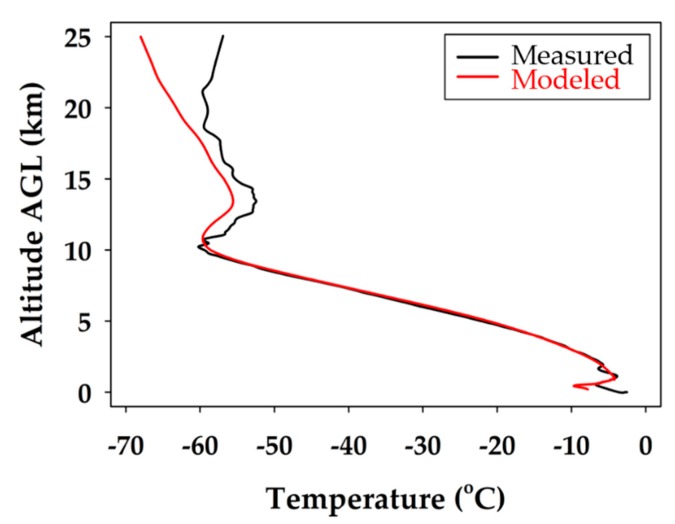
Comparison of modeled temperature to measured atmospheric temperature for the unmanned glider’s descent. The black line represents the measured data and the red line corresponds to the extrapolated WRF modeling data.

**Figure 9:**
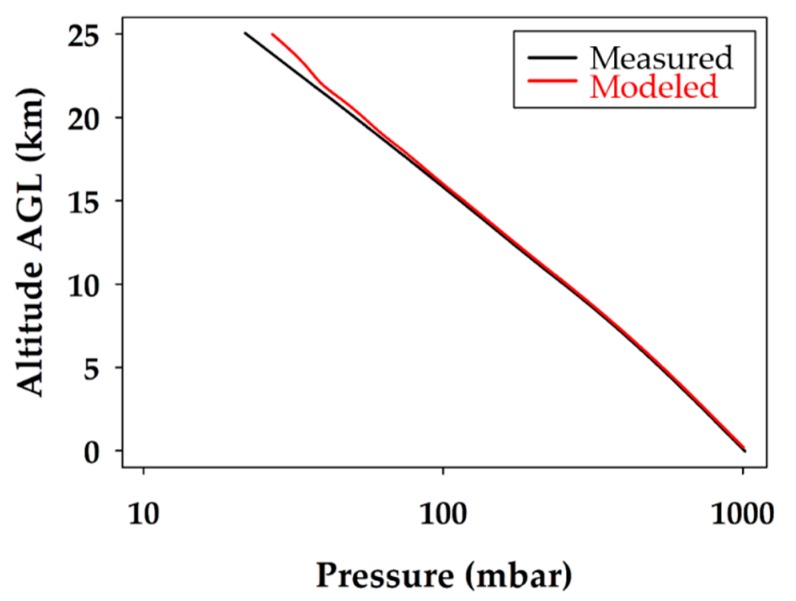
Comparison of modeled and measured atmospheric pressure for the unmanned glider’s descent. The black line represents the measured data and the red line corresponds to the extrapolated WRF modeling data.

**Figure 10:**
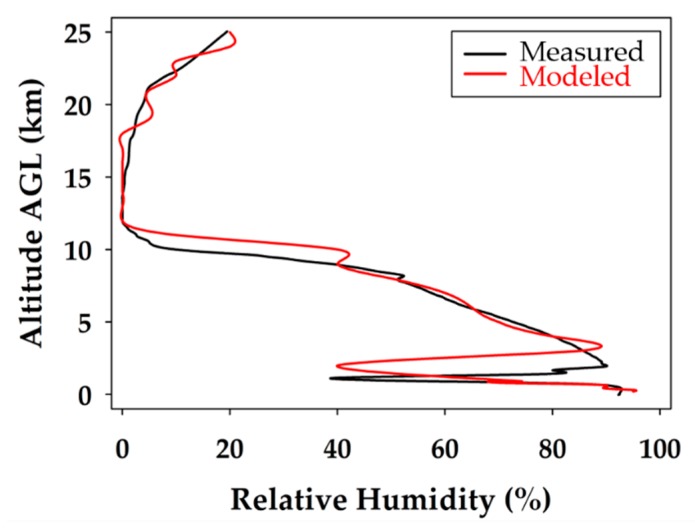
Comparison of modeled and measured relative humidity for the unmanned glider’s descent. The black line represents the measured data and the red line corresponds to the extrapolated WRF modeling data.

The temperature data for the measured and modeled data were plotted against one another for the descent profile in Figure 8. The atmospheric pressure profiles, plotted together on a logarithmic pressure scale, are illustrated in Figure 9. The relative humidity data sets for the measured and modeled data are provided in Figure 10. A two-tailed T test was used to compare the model and measurement data sets, both with different variances, to validate (or reject) the temperature, relative humidity, and pressure predictions. A *p*-value of 0.05 was used as the threshold to determine if a modeled data point was significantly different to the measured value. This was done for the WRF predicted values throughout the entire descent profile.

In Figure 8, both the measured and modeled profiles agree on the atmospheric lapse rate through the troposphere. The profiles both show a temperature inversion at the tropopause inversion at 10 km, and both mirror the same temperature trend up to 18 km. It is apparent that the temperature measured was slightly warmer than the modeled temperature throughout the stratospheric portion of the flight. The temperatures are within a reasonable error for the profile up to the tropopause temperature inversion, but then divert more significantly at 20 km. It shows that the model expects the temperature to continue to decrease with altitude, whereas the measured data show a more stable stratosphere. Above the tropopause, the deviation grew to its maximum when the difference was about 7 °C at 25 km.

From the temperature measurements, it was found that the WRF model predictions were completely accurate up to 20.0 km. It was also determined that 52% and 82% of WRF modeled temperatures are within ±1 and ±2 °C of the measured values, respectively. Therefore, the WRF modeled temperatures are accepted and validated up to 20.0 km. Indeed, the observed temperature deviation could be caused by the lower model accuracy above the tropopause, due to the limited input data available to forecast it. More specifically, the WRF model uses a traditional configuration with 51 vertical grid points distributed in a way to provide higher resolution near the surface but lower quality forecasts ≥10 km from using only 10 grid points above this altitude [12,31]. In other words, the WRF model skews the forecast favoring the lower altitudes and, as a result, it loses accuracy in the predictions at the higher altitudes.

The expected atmospheric pressure profile, plotted on a logarithmic pressure scale in Figure 9, is linear as a function of altitude. Both the modeled and measured profiles agree with great confidence and the lines overlap throughout most of the course of the unmanned glider’s descent. This proves that the model accurately predicted the atmospheric pressure from the surface to 23 km. It also demonstrated the robustness of the unmanned glider’s pumped static port inlet design and its ability to regulate airflow, even at ground speeds in excess of 70 m s^−1^. It was determined that WRF predictions of pressure up to 23.0 km were validated with a 95% confidence level, and that 82% of the modeled pressure data were within ±1 mbar of the measured data. As a result, there is great confidence in the model and the unmanned glider’s ability to accurately measure atmospheric pressure profiles.

When considering the measured relative humidity during the unmanned glider’s descent profile, shown in Figure 10, it is realized that the data match remarkably well with the modeled data throughout the entire profile. The measured data show slightly less moisture stratification, but the major features, and certainly the trends, are the same. This is demonstrated by the closely overlapping lines of modeled and measured data observed throughout the descent profile. The most significant break in the cloud layers, at approximately 2 km, is clearly seen at nearly the same altitude for the measured and modeled data. The discrepancy could be a slight difference in time or location, which produced a small error in the measured and modeled heights of the cloud ceiling above the boundary layer. The results show that all the WRF modeled relative humidity values are acceptable and validated up to 25.0 km with a 95% confidence level. In addition, 88% of the relative humidity predictions were within ±5% of the physical measurements up to 25 km, indicating an outstanding performance by the WRF model, which is validated throughout the entire descent profile.

## 4. Conclusions

Society and businesses are becoming more vulnerable to localized weather (sub 1000 m resolution or higher) events as technical advances in automated vehicles become integrated into routine personal and business activities. Unmanned aerial and ground vehicles are emerging today under controlled test environments. These technologies have some significant weather vulnerabilities that require more precise and accurate predictions to ensure safe and effective ubiquitous operations. WRF-generated predictions provide a solution for localized weather prediction to support future routine operation of automated air and ground vehicles. Improvements in localized WRF predictions will depend on higher resolution observational data sets, especially in the boundary layer. The emergence of commercial weather sensing platforms, such as small satellites, ground-based profilers, and UAV-carrying weather sensors, hold great promise for improving localized WRF predictions. A comprehensive weather sensing strategy that improves localized WRF must include all three observation platforms. Of the three platforms discussed, UAV observations are the only in situ observations available to calibrate the others and allow for accurate verification and validation of localized WRF predictions. This experiment demonstrated the value of in-situ sUAS observations in gaining high altitude weather data sets and in verifying and validating a localized WRF prediction executed in a weather data-sparse location. The results demonstrate the validity of sUAS in situ observations for scientific research and numerical weather prediction validation and verification, and further experimentation. At present, the work does not attempt to apply a data assimilation methodology based on the sUAS data collected. The next question requiring experimentation is whether UAVs carrying weather sensors can affordably scale into a more persistent surveillance strategy to improve weather observation density and quality in the boundary layer where UAVs fly. Collecting sUAS weather observations, especially in high traffic UAV areas, can improve detection and prediction of localized winds and turbulence that impact UAV safety and performance for routine beyond-visual-line-of-sight (BVLOS) flight operations.

## Figures and Tables

**Table 1:** Operational dynamical downscaling weather research and forecasting (WRF) model configuration.

Configuration\Domain	9 km	3 km	1 km
Grid points 1	109 × 109	145 × 145	250 × 250
Vertical levels	50	50	50
Time step	54 s	27 s	9 s
Nesting	No	Yes	Yes
Radiation (shortwave)	RRTMG ^a^ (each 10 min)		
Radiation (longwave)	RRTMG ^a^ (each 10 min)		
Planetary Boundary Layer (PBL)	MM5 ^b^		
Microphysics	WSM6 ^c^		
Surface	Noah Land Surface		
Turbulence	Horizontal Smagorinsky 1st order		

^a^ RRTMG: Rapid Radiative Transfer Model for General Circulation Models [28]; ^b^ MM5: Fifth-generation Mesoscale Model [29]; ^c^ WSM6: WRF single-moment 6-class microphysics scheme [30].

## Abbreviations

ABL	atmospheric boundary layer
AGL	above ground level
ARW	advanced research weather research and forecasting
BVLOS	beyond visual line of sight
GFS	global forecast system
GH	Global Hawk
GPU	graphical processing unit
NCL	NCAR command language
NTC	negative temperature coefficient
NWP	numerical weather prediction
SMARTSonde	Small Multifunction Autonomous Research and Teacher Sonde
sUAS	small unmanned aerial system
SUMO	Small Unmanned Meteorological Observer
UAV	unmanned aerial vehicle
UTC	universal time coordinated
WRF	weather research and forecasting (WRF)
